# Insensibility to Pain

**Published:** 1897-12

**Authors:** 


AMERICAN JOURNAL

—OF—

DENTAL SCIENCE.

VOL. XXXI. BALTIMORE, DECEMBER, 1897. No. 8.

ARTICLE I.

### INSENSIBILITY TO PAIN.

An interesting point has been brought out in a discussion of the dental section of a medical ass ociation on cataphoresis. Cataphoresis is the most modern method of causing the tooth and gums or any part of the body to become insensible to pain. The ordinary method of its exhibition is to saturate a piece of cotton with cocaine and apply the cotton containing electrode to the part to be influenced, a weak electric current being turned on in the meantime. In from one and a half to ten minutes the part becomes "obtunded," or benumed.

A dentist who took part in the discussion said that he had held back—much to the surprise of some of his professional brethern—from the active recognition of cataphoresis in dentistry, and he would now enlighten the association as to his reasons for doing so. The extraction of teeth by electricity was common as far back as 1856, but the real cause of any merit in that method was that the shock produced at the instant the forceps were applied produced a diversion of the will force by causing a sudden and violent inhalation into the lungs. While the lungs remained inflated the effect was excellent, for the senses were for the instant submerged or subjugated. He discovered that the actual insensibility to pain arose from the sudden inhalation of air, and thenceforth abandoned the use of electricity and depends upon rapid breathing alone. In conclusion, he said to his confreres: "You now know why I abandoned electricity for obtunding sensitive dentine and for extracting, for this revelation of how nature relives gave me the clue to the brighter step which dentists have been slow to recognize as a fact. Had you done so, then you would not today be looking for any other agent in most of the cases that it is our lot to have."

Subsequently another practitioner stated that he had made frequent use of the rapid-breathing method to obtund pain for some 16 years, and he thought if the real effort of rapid breathing were better understood it might have a wider application than in dental practice. There are many ways in which a state of disordination of the nerves of feeling may be produced so that they will not carry the sensation of pain to the brain, and thus produce anæsthesia. The way quick breathing acts seems to be by diverting the attention from the operation to the effort to breathe quickly. In hipnotism sleep is caused by occupying the patient's attention, and thus disordering the centres of mentality and the centres of sensation, so that they do not work together. A case is reported of tumor operation in which the man was made to hold the surgeon's hand and maintain constant pressure. This will make anyone tired, and the effort diverted the attention of the patient from the pain of the operation so fully that the tumor was removed by ligation without any pain being felt, and without the loss of consciousness at any time. All that seems necessary is to fix the thoughts of the patient closely on something else while the pain lasts. This answers very well when the tooth can be drawn instantly, but if this or any other operation has to be prolonged, there is no question that cataphoresis is the surest, simplest, and best method known of securing insensibility to pain.—*Pittsburg Dispatch.*

